# Non-destructive identification of commercial jerky types based on multi-band hyperspectral imaging with machine learning

**DOI:** 10.1016/j.fochx.2025.102293

**Published:** 2025-02-19

**Authors:** Yuanxi Han, Liang Li, Siyuan Jiang, Pengpeng Sun, Wenliang Wu, Zhendong Liu

**Affiliations:** a Food Science College, Xizang Agriculture & Animal Husbandry University, R&D Center of Agricultural Products with Xizang Plateau Characteristics, The Provincial and Ministerial Co-founded Collaborative Innovation Center for R&D in Xizang Characteristic Agricultural and Animal Husbandry Resources, Nyingchi 860000, China; b College of Information Engineering, Northwest A&F University, Shaanxi, Xianyang, 712100, China

**Keywords:** Commodity jerky, Hyperspectral imaging, Machine learning, Spectral band optimization, Type identification

## Abstract

Commercial jerky counterfeiting is widespread in the market. This study combined visible-near-infrared and short-wave-near-infrared hyperspectral imaging along with multiple machine learning algorithms for non-destructive identification of five types of commercial jerky products, and explored the impact of different spectral bands, algorithm selection, and optimization methods on identification performance. After data preprocessing, all models' accuracies and stability improved. Specifically, the logistic regression model was best for jerky identification, with 85.78 %–100.00 % accuracy. With hyperparameter optimization, Support Vector Machine with linear kernel had highest accuracy (89.29 % and 95.29 % in two bands). Additionally, the artificial neural network with the hyperbolic tangent activation function had optimal training performance, exceeding 90.00 % accuracy. The findings demonstrate short-wave-near-infrared hyperspectral imaging combined with linear models (logistic regression and Support Vector Machine with linear kernel parameter settings) is better for identifying the types of jerky. This study provides reference for the band, model selection, and optimization of jerky type identification.

## Highlights

• Non-destructive rapid identification of commercial jerky types using Vis-NIR-HSI and SWIR-HSI.

• Explored the impact of spectral bands, algorithms, and optimizations on jerky type identification.

• SWIR-HSI combined with nonlinear models can better identify among jerky types.

## 1 Introduction

Meat is an essential component of the human diet, rich in vital nutrients such as protein, fat, and vitamins. To prolong the storage time of fresh meat, individuals employed traditional pickling and dehydration techniques to make jerky in the past, making it one of the oldest meat products (Yang, Hwang, Joo, & Park, 2009). With advancements in food processing technology, jerky has gradually evolved into a popular snack and has captured a considerable share of the consumer market (Luo et al., 2020).

Jerky is produced from a single kind of livestock or poultry meat through processes including trimming, curing, and drying. Spices and functional additives are frequently added in production to enhance the flavor and nutritional value of jerky (Konieczny, Stangierski, & Kijowski, 2007). The types of jerky are diverse, with ingredients ranging from common domesticated animals (such as cows, pigs, or ducks) to rare domesticated species (including yaks or deer). However, discrepancies in the availability and feeding costs of different jerky ingredients result in a significant gap in their market value (Wang et al., 2016). As market demand persists in growing, several merchants exploit the price gaps in jerky and pursue unjust profits through actions like employing deceptive labels or peddling counterfeit products. These fraudulent practices involve, but are not limited to, substituting pork or duck jerky for beef jerky (Li et al., 2023), and even using these jerky products as higher-priced yak jerky (Wang et al., 2016). According to statistical data from Li et al. (Li et al., 2023), from 2012 to 2021, jerky had emerged as one of the primary targets for counterfeit substitution in the Chinese market, ranking third in the frequency of false substitution after fresh mutton and beef. Additionally, research conducted by Wang et al. (Wang et al., 2016) suggested that around 70 % of yak jerky products available on the market were counterfeit. It is essential to note that post-curing and processing, the flavor and appearance of fresh meat from various species commonly converge, rendering it challenging for consumers to distinguish the authenticity of the product when purchasing jerky.

Ever since food fraud incidents aroused public concern, meat and meat products are one of the high-incidence domains of food fraud cases (Wang et al., 2016), seriously infringing on consumer rights and posing a threat to public health (Muslim, Abbood, Hammood, & Azooz, 2024). This trend has promoted the rapid development of meat authenticity techniques. To date, researchers have conducted investigations on the authenticity of jerky products employing various techniques, including traditional physicochemical testing, nucleic acid analysis techniques, and spectral analysis techniques. In detail, YANG et al. (Yang et al., 2009) compared the physicochemical indices of between beef jerky with pork jerky, found that pork jerk had higher brightness, shear force, and 2-thiobarbituric acid value than with those in beef jerky. By application of nucleic acid analysis techniques, WANG et al. (Wang et al., 2016) and KIM (Kim & Kim, 2017) identified commercial beef jerky and yak jerky. In terms of spectral analysis techniques, Mamani-Linares et al. (Mamani-Linares, Alomar, & Gallo, 2012) identified jerky types from beef, horse, and alpaca using near-infrared spectroscopy. Despite the aforementioned studies have shown physicochemical test and nucleic acid method could be successfully applied to identify jerky authenticity, these techniques still need complicated sample pretreatment and destructive testing procedures. Moreover, the thermal processing involved in jerky production may lead to DNA degradation (Kim & Kim, 2017). Although near-infrared spectroscopy effectively solves above problems well, single-point detection is insufficient for characterization of jerky samples information so comprehensively (Jiang, Cheng, & Shi, 2020).

As an emerging technoloy, hyperspectral imaging combines spectroscopy with computer vision, and has non-destructive, rapidity and real-time capabilities. It not only provides spatial and spectral information about samples but also effectively overcomes the limitations of insufficient accuracy in single-point detection (Jiang et al., 2020). In combination with machine learning, this technique can be extracted from hyperspectral multidimensional data and realize sample classification or prediction through self-learning Capabilities (Saha & Manickavasagan, 2021). This has led to a broader application of hyperspectral imaging technology in meat assessment, where it is regarded as a promising evaluation tool (Jia, van Ruth, Scollan, & Koidis, 2022). Based on this technique, significant progress has been made in identifying species (Al-Sarayreh, Reis, Yan, & Klette, 2020), adulteration (Yang et al., 2023), and assessing feeding conditions (Xiong, Sun, Pu, Zhu, & Luo, 2015) of fresh meat. However, no research has been reported concerning commercial jerky.

This study examined five types of commercially available jerky that are commonly conflated: chicken jerky, yak jerky, beef jerky, duck jerky, and pork jerky. Hyperspectral imaging equipment in the visible and near-infrared (Vis-NIR) and short-wave infrared (SWIR) ranges were employed to acquire reflectance spectra of these jerky products. Various machine learning algorithms, Support Vector Machine (SVM), Artificial Neural Network (ANN), Naive Bayes (NB), Logistic Regression (LR), Decision Tree (DT), and Random Forest (RT), were utilized to achieve efficiently identify distinct types of jerky, with the expectation of establishing a scientific foundation for the authenticity identification of jerky products.

## 2 Materials and methods

### 2.1 Sample collection and preparation

In this study, five types of commercial meat jerky (5 × 30 = 150) were selected, including chicken jerky (*n* = 30), yak jerky (n = 30), beef jerky (n = 30), duck jerky (n = 30), and pork jerky (n = 30). To ensure the consistency and authenticity of the samples, all were uniformly processed and produced by Tibet Tianhu Herdsman Agriculture and Animal Husbandry Science and Technology Development Company Limited, adhering to the standards set forth in NY/T2782–2015 Technical Specification for the Processing of Air-dried Meat. The raw materials—chicken breast, yak hind leg, cow hind leg, duck breast, and pig hind leg—were sourced from the Lhasa Livestock and Poultry Market and transported at −18 °C to the processing facility of Tibet Tianhu Herdsman Agriculture and Animal Husbandry Technology Development Co., Ltd.

The thawed and cleaned raw meat was trimmed into 15 cm × 4 cm × 3 cm strip-shaped pieces. The basic marinade prepared by Xizang Tianhu grazers was added for marination, along with the basic seasoning formula (comprising salt, sugar, chili pepper, pepper powder, monosodium glutamate, and spices). The marination process was conducted at 4 °C for 24 h. Subsequently, the marinated meat strips were hung on an air-drying rack and naturally air-dried for 72 h at 25 °C. The air-dried meat was then baked at 180 °C for 3 min, spread out to cool, sealed in bags, and subjected to microwave sterilization for 5 min. The final product was stored at room temperature for later use.

### 2.2 Hyperspectral image acquisition

**Fig. 1:**
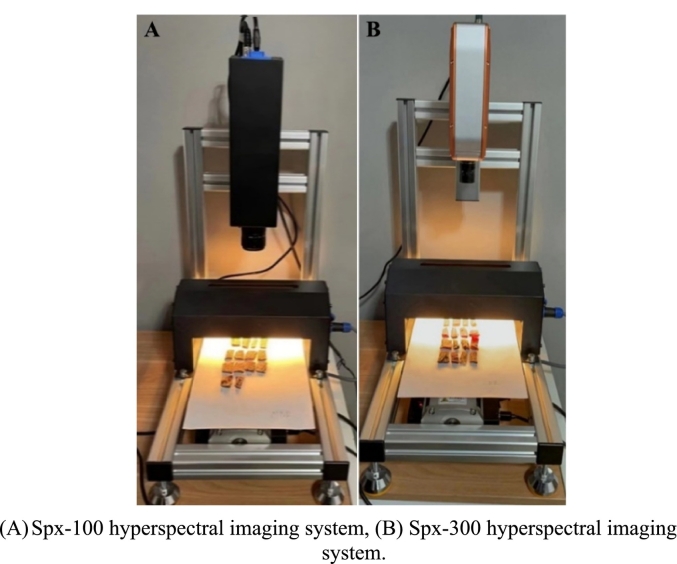
External push-scan hyperspectral imaging systems.

Vis-NIR hyperspectral data acquisition was conducted using the Spx-100 hyperspectral imaging system (Zhejiang Yixiang Technology Co., Ltd., Zhejiang Province, Wenzhou City, Lucheng District, China), which adopted external push-scan technology to collect reflectance mode images in the wavelength range of 400–1000 nm. This system has 1200 spectral channels, a wavelength resolution of 2.5 nm, a full-spectrum acquisition speed ≤128 FPS, and a spatial resolution of 1920 × 1080 pixels for the collected data. Meanwhile, SWIR hyperspectral data acquisition was carried out with the Spx-300 hyperspectral imaging system (Zhejiang Yixiang Technology Co., Ltd., Zhejiang Province, Wenzhou City, Lucheng District, China). Similar to the Spx-100, this system adopted external push-scan technology and performed reflectance mode image acquisition within the wavelength range of 900–1700 nm. The Spx-300 has 254 spectral channels, a wavelength resolution of 8.0 nm and can acquire full spectrum data at ≤200 FPS along with one sample per hour for spatial resolution 320 × 320 pixels captured resultants datasets. The configurations of the Spx-100 and Spx-300 hyperspectral imaging systems are shown in Fig. 1.

The jerky samples were arranged in a sequence on the tray during the specific acquisition, which was placed at mobile control platform for collection. The temperature and humidity in the detection environment were maintained at 25 ± 5 °C and remained constant. To minimize the interference from ambient light, stringent light-shielding measures were implemented to ensure that the hyperspectral camera operates in a completely dark environment. Before acquisition, the imaging system was activated for 30 min of preheating to maintain optimal image quality. The raw images (I_RAW_) obtained underwent calibration using dark reference images (I_D_) and white reference images (I_W_) to mitigate noise signals resulting from dark current effects. The specific calibration formula is shown in Formula (1) (Jin et al., 2023).

(1) $$I=\frac{I_{\mathrm{RAW}}-I_{D}}{I_{w}-I_{D}}$$

### 2.3 The analysis of the data

#### 2.3.1 Data analysis steps

**Fig. 2:**
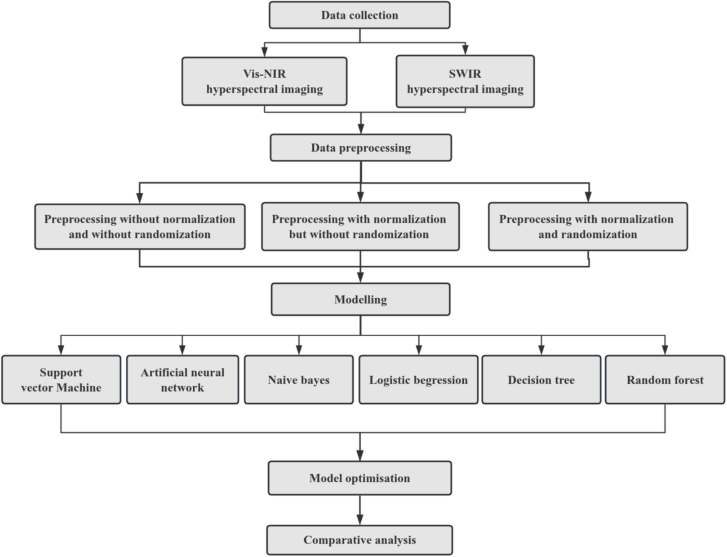
Data analysis steps.

In order to realize the research objective of combining spectral data with machine learning for identifying types of jerky and exploring the factors that influencing classification performance. The following steps were proposed for the research work, as illustrated in Fig. 2.

Following the capturing of hyperspectral data of jerky samples by Vis-NIR hyperspectral system (400–1000 nm) and the SWIR hyperspectra system (700–1900 nm), data preprocessing for the raw hyperspectral data was an effective step to reduce interference from instrument-induced variations, such as random noise and spectral shifts. Normalization preprocessing was a commonly used method in metabolomics, aimed at adjusting data ranges and reducing batch effects. By transforming the data to a uniform scale, it eliminated discrepancies in spectral intensity and instrumental influences, thereby enhancing data comparability and analytical accuracy (Misra, 2020). Randomization preprocessing aimed to minimize the order dependence in data by randomizing data labels or sample allocation. This method not only assisted in balancing the class distribution within the dataset, but also improved the overall performance of the model (Pham & Smeulders, 2008). To explore the impact of preprocessing methods on the performance of identification models, we conducted a comparative analysis including three scenarios: no normalization with no randomization, normalization without randomization, and normalization with randomization. Due to the balanced quantity of various samles, the obtained data were divided into training set and testing set with a ratio of 9:1 (Rachineni et al., 2022), and a fixed random seed of 42 was set to ensure the reproducibility of the dataset partitioning. Among them, the training set was used for model training to ensure that the model can effectively learn the features and underlying patterns within the data, while the testing set was used to evaluate the classification accuracy of the recognition model for unknown data, providing a realistic measure of the model's actual performance (Kang & Oh, 2020).

To explore the applicability of multiple machine learning algorithms in solving the jerky identification problem based on spectral data, six widely used algorithms for classification tasks: SVM, ANN, NB, LR, DT and RF, were selected. The objectives of this part of the work were as follows: (1) Comparison and analysis of jerky identification based on two spectral data sets with different bands. (2) Comparative analysis of jerky identification effect based on different algorithms. (3) Detailed comparison of the accuracy of each algorithm in specific identified samples.

Finally, to optimize jerky classification models, parameters for each machine learning algorithm were adjusted. Subsequently, the classification performance of the established recognition models on both the training and testing sets was compared and analyzed.

#### 2.3.2 Model establishment

SVM: SVM is a supervised learning method primarily used for classification and regression tasks, noted for its strong generalization ability. The fundamental concept involves mapping data into a high-dimensional space using a kernel function to facilitate the construction of an optimal classification hyperplane in that space. The choice of different kernel functions determines the nature and dimensionality of the high-dimensional space to which the data is mapped, enabling SVM to effectively address a variety of complex data relationships, including both linear and nonlinear patterns. One of the most commonly used types of kernel function is the radial basis function (You, Kim, Lee, Choi, & Ieee., 2017).

ANN: ANN is a machine learning algorithm that simulates the information processing mechanisms of the human brain. It has been shown to excel in handling highly nonlinear classification tasks and multi-dimensional data analysis in the food industry (Raj & Dash, 2022). The ANN typically consists of an input layer, hidden layers, and an output layer, where the number of units in the hidden layers affects the network's ability to process nonlinearity (Taner, Öztekin, Tekgüler, Sauk, & Duran, 2018). In this study, a fully connected neural network with hidden layers was employed to perform classification tasks. Specifically, the input layer comprises 300 and 254 neurons, corresponding to the effective features extracted from two distinct spectral bands. The architecture includes two hidden layers with 128 and 64 neurons, respectively, and an output layer consisting of 5 neurons. This configuration enables the network to utilize reflectance data as input features, perform nonlinear transformations and pattern recognition in the hidden layers, and ultimately predict the class labels of jerky samples in the output layer.

NB: NB is a supervised learning method based on Bayes' theore. It evaluates evaluates the likelihood of a sample belonging to a certain category based on the probability values of each feature. (Saha & Manickavasagan, 2021). Due to its simplicity, efficiency, and immunity to the curse of dimensionality (Syazwani, Asraf, Amin, & Dalil, 2022), NB is particularly suitable for multivariate spectral data and small-sample datasets. In this study, NB assumed that the hyperspectral reflectance of jerky samples at each wavelength was normally distributed, and the hyperspectral reflectance at different wavelengths was assumed to be probabilistically independent of each other. When identifying the types of jerky, it was only necessary to repeatedly calculate the conditional probability of each type of jerky and combine it with Bayesian formula to achieve the identification of jerky types.

LR: LR is a supervised learning method based on the linear regression model (Wu et al., 2019). It is best suited for binary identification tasks and on linearly separable datasets. To further improve the model performance for multi-classification of jerky, this study introduced the ‘Softmax’ activation function to enhance model performance. Additionally, to reduce the potential for overfitting caused by the imbalance between the number of samples and the number of features in logistic regression (Feyzioglu & Taspinar, 2023), the dual parameters could be adjusted to optimize the model.

DT: DT is a supervised learning method based on a tree structure that consist of three parts: the root node, non-leaf node (branch), and leaf node (Du et al., 2019). The construction process followed a hierarchical decision mechanism from the root node to the leaf node: the root node represented the entire dataset of samples (which could be either the training set or the test set), and each non-leaf node divided its subset based on optimal features. The division process eventually halted at the leaf node, which corresponded to the label of the jerky type and represented the identification result of the decision tree. The splitting at the non-leaf node was based on criteria such as entropy or the ‘Gini’ index, with the selection of optimal features aimed at improving classification accuracy (Aqeel, Sohaib, Iqbal, Rehman, & Rustam, 2024).

RF: RF is a supervised learning method based on an ensemble of DTs (Saberioon et al., 2018), suitable for handling large and nonlinear datasets (de Santana, Neto, & Poppi, 2019). In multiclass identification tasks, RF votes or averages the prediction results from each Decision Tree (DT), based on randomly sampled attributes and feature information. Compared to a single DT, RF generally exhibits superior generalization performance and a greater ability to combat overfitting.

Upon initial establishment of the models, the default hyperparameter settings for each algorithm were as follows: the SVM employed the ‘Radial Basis Function (RBF)’ kernel; the ANN utilized a four-layer structure comprising two hidden layers, with the hidden layers employing the ‘Rectified Linear Unit’ (ReLU) activation function, and the output layer utilizing the ‘Softmax function’; the LR set the Solver parameter to the ‘Limited-memory Broyden–Fletcher–Goldfarb–Shanno’ (L-BFGS) algorithm, with the dual parameter set to False; and for the DT and RF algorithms, the default split criterion was the ‘Gini’ index.

#### 2.3.3 Model establishment

Since the number of sample strips of the five types of jerky was balanced, this study employed a 10 × 10-fold cross-validation approach to evaluate the identification performance of the model based on the average accuracy. Additionally, the average precision, average recall, and macro-averaged F1 score were incorporated to ensure the reliability and stability of the evaluation results. The definitions of these evaluation metrics were described as follows:

(2) $${\text{Accuracy}}_{k}^{\left(i\right)}=\frac{{\mathrm{TP}}_{k}^{\left(i\right)}+{\mathrm{TN}}_{k}^{\left(i\right)}}{{\mathrm{TP}}_{k}^{\left(i\right)}+{\mathrm{TN}}_{k}^{\left(i\right)}+{\mathrm{FP}}_{k}^{\left(i\right)}+{\mathrm{FN}}_{k}^{\left(i\right)}}$$

(3) $${\text{Accuracy}}_{10\times 10-\text{fold}}=\frac{1}{10}\sum_{i=1}^{10}\left(\frac{1}{10}\sum_{k=1}^{10}{\text{Accurcy}}_{k}^{\left(i\right)}\right)$$

(4) $${\text{Precision}}_{k}^{\left(i\right)}=\frac{{\mathrm{TP}}_{k}^{\left(i\right)}}{{\mathrm{TP}}_{k}^{\left(i\right)}+{\mathrm{FP}}_{k}^{\left(i\right)}}$$

(5) $${\text{Precision}}_{10\times 10-\text{fold}}=\frac{1}{10}\sum_{i=1}^{10}\left(\frac{1}{10}\sum_{k=1}^{10}{\text{Precision}}_{k}^{\left(i\right)}\right)$$

(6) $${\text{Precision}}_{k}^{\left(i\right)}=\frac{{\mathrm{TP}}_{k}^{\left(i\right)}}{{\mathrm{TP}}_{k}^{\left(i\right)}+{\mathrm{FN}}_{k}^{\left(i\right)}}$$

(7) $${\text{Recall}}_{10\times 10-\text{fold}}=\frac{1}{10}\sum_{i=1}^{10}\left(\frac{1}{10}\sum_{k=1}^{10}{\text{Recall}}_{k}^{\left(i\right)}\right)$$

(8) $$F1\;{\text{Score}}_{k}^{\left(i\right)}=2\times \frac{{\text{Precision}}_{k}^{\left(i\right)}\times {\text{Recall}}_{k}^{\left(i\right)}}{{\text{Precision}}_{k}^{\left(i\right)}+{\text{Recall}}_{k}^{\left(i\right)}}$$

(9) $$F1\;{\text{Score}}_{10\times 10-\text{fold}}=\frac{1}{10}\sum_{i=1}^{10}\left(\frac{1}{10}\sum_{k=1}^{10}F1\;{\text{Score}}_{k}^{\left(i\right)}\right)$$

In these formulas, *i* denoted the outer loop of the *i*-th 10-fold cross-validation, with a range of 1 ≤ *i* ≤ 10. The parameter *k* represented the inner loop of the *k*-th fold within each 10-fold cross-validation, with a range of 1 ≤ *k* ≤ 10. For each fold k in repetition i: ${\mathrm{TP}}_{k}^{\left(i\right)}$=True positives; ${\mathrm{FP}}_{k}^{\left(i\right)}$=False positives; ${\mathrm{TN}}_{k}^{\left(i\right)}$=True Negatives.

### 2.4 Software

In this study, segmentation of hyperspectral images and selection of Regions of Interest (ROIs) were conducted using SpecimINSIGHT software (Specim, Spectral Imaging Ltd., Oulu, Finland). The preprocessing of hyperspectral data and model establishment were implemented using the Python library for the Scikit-learn platform. The total reflection spectrum images of different bands were analyzed using the original 2021 software (Origin Lab Corporation, Roundhouse Plaza, Northampton, MA, USA).

## 3 Results and analysis

### 3.1 Spectral analysis of jerky samples

**Fig. 3:**
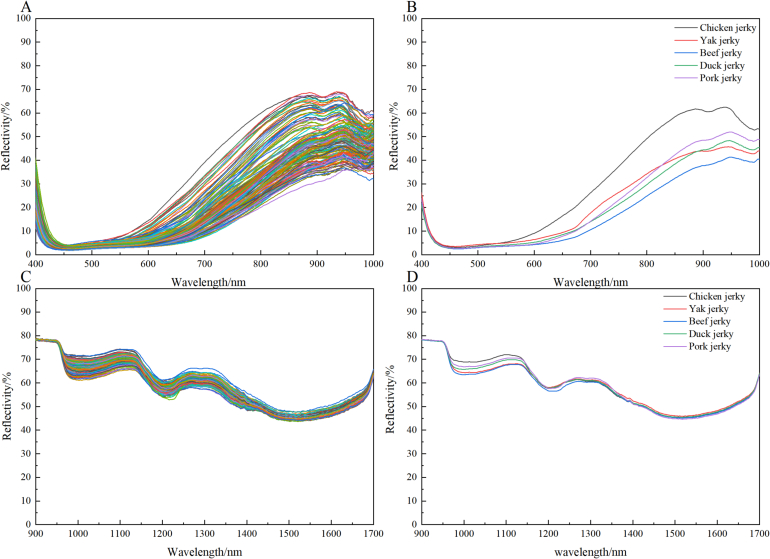
Reflectance spectral images of jerky samples at different wavelengths.

The total reflectance spectra images across several bands were shown in Fig. 3, along with the average reflectance spectra derived from five types of jerky samples. The hyperspectral matrices 150× 300 and 150× 254 were acquired in the Vis-NIR and SWIR bands, respectively. In the visible spectrum range of 400–700 nm, the spectral data mainly reflected the color information of the samples. The average reflectance curves of the different types of jerky overlapped in the green-blue range (400–550 nm) with low reflectance values, indicating high absorbance. In the yellow range (550–600 nm) and the red range (600–700 nm), the reflectance curves began to diverge, showing an increase in reflectance. This variation in reflectance illustrated the color differences among the different types of jerky (Zhang, Jiang, & Wang, 2019), particularly in the red range, where the reflectance of chicken jerky was significantly higher than that of the other four types, leading to a perceptually redder appearance. In the near-infrared spectral range of 700–1700 nm, absorption bands appeaerd at 970 nm and 1400 nm. The absorption bands at 970 nm and 1400 nm corresponded to the second overtone of O—H stretching vibrations, primarily associated with the moisture content of the samples (Li et al., 2021). The absorption peak at 1200 nm was mainly related to aliphatic C—H groups associated with the second harmonic (Dashti et al., 2023), and the absorption peak at 1450 nm corresponded to the first harmonic of N—H vibrations (Zheng, Chen, Li, & Zhang, 2023).

### 3.2 Comparison of classification performance among various preprocessing techniques and machine learning algorithms under multispectral conditions

Data normalization was a crucial preprocessing step that had been proven effective in reducing systematic noise in omics datasets (Vu, Riekeberg, Qiu, & Powers, 2018). The analysis of the original spectra of several jerky varieties indicated that their reflectance spectral trends were similar and highly overlapping. Although some distinguishable features exist in the raw reflectance spectra, these features were often obscured by noise and other interference factors, and different features exhibited varying dimensions and value ranges. Additionally, the interference factors present in the data may have lead to model estimation bias. Normalization reduces interferences and unifies the data range, while random permutation introduces diversity to enhance model performance and generalization. Table 1, Table 2 delineated the classification results obtained from processing the training and testing datasets in three different ways: normalization without randomization, normalization with randomization, and no normalization or randomization.

**Table 1:** Comparison of accuracy of jerky types identification across different preprocessing methods in the Vis-NIR spectral range.

Machine learning algorithms	10 × 10-fold cross-validation re-averaged accuracy	Preprocessing without normalization and without randomization	Preprocessing with normalization and without randomization	Preprocessing with normalization and randomization
SVM	Training accuracy	74.57 %	88.14 %	87.99 %
	Test accuracy	72.67 %	54.19 %	79.24 %
	Test precision	73.34 %	44.94 %	86.18 %
	Recall	77.24 %	35.73 %	80.48 %
	F1 score	71.63 %	34.14 %	80.43 %
ANN	Training accuracy	68.12 %	87.16 %	87.38 %
	Test accuracy	54.50 %	76.50 %	84.54 %
	Test precision	47.08 %	79.83 %	79.05 %
	Recall	44.35 %	63.14 %	79.13 %
	F1 score	41.63 %	69.57 %	75.99 %
NB	Training accuracy	77.55 %	77.55 %	77.32 %
	Test accuracy	64.19 %	64.19 %	75.81 %
	Test precision	39.29 %	39.29 %	69.61 %
	Recall	33.90 %	33.90 %	71.26 %
	F1 score	32.55 %	32.55 %	67.76 %
LR	Training accuracy	95.52 %	98.20 %	98.11 %
	Test accuracy	65.00 %	71.57 %	91.92 %
	Test precision	65.67 %	64.44 %	89.67 %
	Recall	62.92 %	56.94 %	92.55 %
	F1 score	61.23 %	57.68 %	89.52 %
DT	Training accuracy	100.00 %	100.00 %	100.00 %
	Test accuracy	58.06 %	56.91 %	69.65 %
	Test precision	39.67 %	38.01 %	70.00 %
	Recall	34.29	32.31 %	72.20 %
	F1 score	31.36 %	30.01 %	66.96 %
RF	Training accuracy	100.00 %	100.00 %	100.00 %
	Test accuracy	58.35 %	58.38 %	73.48 %
	Test precision	99.00 %	98.92 %	80.76 %
	Recall	60.81 %	58.14 %	77.00 %
	F1 score	70.35 %	67.08 %	80.13 %

**Table 2:** Comparison of accuracy of jerky types identification across different preprocessing methods in the SWIR spectral range.

Machine learning algorithms	10 × 10-fold cross-validation re-averaged accuracy	Preprocessing without normalization and without randomization	Preprocessing with normalization and without randomization	Preprocessing with normalization and randomization
SVM	Training accuracy	61.81 %	92.17 %	91.83 %
	Test accuracy	34.24 %	66.48 %	81.12 %
	Test precision	37.05 %	43.25 %	97.17 %
	Recall	47.20 %	39.36 %	83.86 %
	F1 score	35.19 %	37.09 %	84.26 %
ANN	Training accuracy	30.69 %	91.26 %	91.21 %
	Test accuracy	20.01 %	79.46 %	88.85 %
	Test precision	8.61 %	79.73 %	80.90 %
	Recall	6.95 %	64.52 %	84.33 %
	F1 score	5.11 %	69.00 %	80.30 %
NB	Training accuracy	77.11 %	77.11 %	76.51 %
	Test accuracy	62.57 %	62.57 %	71.49 %
	Test precision	45.97 %	45.97 %	70.17 %
	Recall	44.70 %	44.70 %	70.53 %
	F1 score	41.43 %	41.43 %	67.21 %
LR	Training accuracy	95.08 %	99.40 %	99.31 %
	Test accuracy	81.14 %	78.52 %	94.07 %
	Test precision	72.50 %	67.07 %	91.96 %
	Recall	76.93 %	69.79 %	89.43 %
	F1 score	73.11 %	65.14 %	89.58 %
DT	Training accuracy	100.00 %	100.00 %	100.00 %
	Test accuracy	50.17 %	50.11 %	60.73 %
	Test precision	31.46 %	32.92 %	66.74 %
	Recall	20.04 %	23.11 %	68.80 %
	F1 score	22.46 %	25.06 %	64.71 %
RF	Training accuracy	100.00 %	100.00 %	100.00 %
	Test accuracy	61.30 %	61.34 %	79.65 %
	Test precision	98.51 %	98.57 %	81.56 %
	Recall	60.43 %	63.81 %	77.00 %
	F1 score	71.31 %	74.90 %	75.45 %

Analysis showed that for spectral data from the Vis-NIR and SWIR bands, normalization preprocessing enhanced the average accuracy in both training and test sets for the ANN. This indicated that normalization had a positively impacts on these algorithms, enabling it to more effectively handle scale disparities among features and thereby improving model training performance. However, within the default parameters, although normalization increased the training and test accuracy of the SVM and LR algorithms, it had a negligible impact on the prediction accuracy of other algorithms such as RF, DT, and NB, and their generalization ability still required improvement. Overall analysis revealed that when using a combination of normalization and randomization for preprocessing, the average accuracy and performance metrics on the test set for all machine learning algorithms across both bands were superior to those achieved with only normalization or no preprocessing. This suggested that the additional randomness and data diversity introduced by randomization could further enhance the models' dependence on the order of sample data, thereby enhancing their recognition effectiveness and generalization ability. Additionally, among all algorithms, the LR algorithm achieved the highest average accuracy on the test set, at 91.92 %, and exhibited the best overall model performance. While the DT algorithm achieved 100 % average accuracy on the training set, its accuracy on the test set was below 70 %, the lowest among all algorithms.

### 3.3 An investigation into the classification performance of various machine learning algorithms on different jerky types under optimal preprocessing techniques

To further analyze the classification effect of different machine learning algorithms on five types of jerky under normalization and randomization preprocessing, Table 3, Table 4 present the specific results for the Vis-NIR and SWIR spectral bands. The classification performance of different machine learning algorithms across the two spectral bands varies, yet there are commonalities.

**Table 3:** Identification accuracy of various machine learning algorithms in the Vis-NIR spectral range.

Machine learning algorithms	10 × 10-Fold cross-validation re-averaged accuracy	Chicken jerky	Yak jerky	Beef jerky	Duck jerky	Pork jerky
SVM	Training accuracy	98.89 %	95.50 %	87.72 %	69.69 %	88.13 %
	Test accuracy	97.50 %	94.17 %	83.50 %	46.67 %	76.17 %
ANN	Training accuracy	96.12 %	83.65 %	76.18 %	84.80 %	96.15 %
	Test accuracy	93.00 %	80.21 %	73.02 %	81.95 %	94.52 %
NB	Training accuracy	94.31 %	79.94 %	83.08 %	53.97 %	75.28 %
	Test accuracy	93.13 %	79.83 %	83.08 %	52.12 %	70.89 %
LR	Training accuracy	100.00 %	100.00 %	99.44 %	98.32 %	92.80 %
	Test accuracy	100.00 %	100.00 %	87.62 %	85.78 %	86.22 %
DT	Training accuracy	100.00 %	100.00 %	100.00 %	100.00 %	100.00 %
	Test accuracy	90.06 %	89.94 %	56.09 %	44.04 %	68.12 %
RF	Training accuracy	100.00 %	100.00 %	100.00 %	100.00 %	100.00 %
	Test accuracy	97.74 %	78.67 %	82.67 %	49.49 %	58.83 %

**Table 4:** Identification accuracy of various machine learning algorithms in the SWIR spectral range.

Machine learning algorithms	10 × 10-Fold cross-validation re-averaged accuracy	Chicken jerky	Yak jerky	Beef jerky	Duck jerky	Pork jerky
SVM	Training accuracy	98.56 %	91.20 %	84.69 %	92.16 %	92.56 %
	Test accuracy	82.14 %	82.14 %	72.33 %	84.50 %	84.67 %
ANN	Training accuracy	96.65 %	86.16 %	91.95 %	89.17 %	97.12 %
	Test accuracy	94.00 %	83.33 %	88.49 %	85.33 %	93.10 %
NB	Training accuracy	89.06 %	65.18 %	86.42 %	57.93 %	83.96 %
	Test accuracy	80.48 %	59.48 %	81.73 %	54.60 %	81.17 %
LR	Training accuracy	100.00 %	100.00 %	99.87 %	100.00 %	96.67 %
	Test accuracy	100.00 %	97.85 %	86.42 %	92.91 %	93.19 %
DT	Training accuracy	100.00 %	100.00 %	100.00 %	100.00 %	100.00 %
	Test accuracy	91.73 %	51.16 %	48.46 %	61.08 %	51.22 %
RF	Training accuracy	100.00 %	100.00 %	100.00 %	100.00 %	100.00 %
	Test accuracy	97.29 %	65.50 %	78.47 %	71.93 %	85.05 %

Based on the spectral data from the two bands, the LR algorithm achieved classification accuracies exceeding 85.00 % for all jerky types. Among the six algorithms, it demonstrated the highest average accuracy in predicting chicken, yak, and duck jerky. In contrast, the NB algorithm exhibited the lowest average accuracy in identifying the other four types of jerky, excluding beef jerky. For all jerky types, both the DT and RF algorithms attained an average training accuracy of 100 %; however, only a subset of types achieved prediction accuracies above 80.00 % in the testing set.

The above results may be related to the characteristics of the algorithms themselves. For instance, the NB algorithm performs poorly when classifying jerky types, which may be attributed to its assumption of feature independence. In reality, the spectral data may contain latent interaction effects or correlations among features that the NB algorithm finds difficult to learn(Pan, Li, Fu, Chang, & Chen, 2022). On the other hand, despite the excellent performance of the DT and RF algorithms on the training set, the poor performance on the prediction set could be attributed to model complexity and susceptibility to data noise (Arar & Ayan, 2017) (Saha & Manickavasagan, 2021).

The comparative analysis reveals substantial disparities in the performance of various algorithms across different jerky types. In the Vis-NIR spectral band, all algorithms achieve prediction accuracies exceeding 90.00 % for chicken jerky. In yak jerky, the SVM, ANN, LR and DT algorithms all performed better with an accuracy of 100 in LR algorithm. The SVM, NB LR RF algorithms perform well in the identification of beef jerky. Yet in identifying duck and pork jerky, only the ANN and LR algorithms show notable performance, whereas the other algorithms exhibit prediction accuracies below 80 %, with RF achieving the lowest accuracy at 58.63 %. In the SWIR spectral band, the algorithms similarly perform well in classifying chicken jerky. The ANN and LR algorithms exhibit outstanding performance in categorizing yak, beef, and duck jerky, and the SVM also effectively identified between yak and duck jerky. In the classification of pork jerky, the DT algorithm has a relatively poor prediction accuracy of 51.22 %, while the other five algorithms achieve accuracies above 80.00 %.

Notably, under the SWIR spectral band, the six machine learning algorithms generally achieve higher accuracy rates in classifying duck jerky compared to the Vis-NIR band (D. Wu & Sun, 2017). This improvement is likely attributed to the SWIR band providing more detailed information about the protein and fat composition of the jerky, which enhances the differentiation of duck jerky (Dashti et al., 2023). While the LR algorithm shows a slight decline in accuracy for yak and beef jerky test datasets under the SWIR band, overall, this spectral band ensures high training accuracy for the remaining three types of jerky while also improving the accuracy of the prediction datasets. Conversely, under the Vis-NIR band, the training and test dataset accuracy rates for chicken jerky, with the exception of the ANN and DT algorithms, are higher than those achieved under the SWIR band. This analysis indicates the influence and complementary nature of spectral information from different bands on the differentiation of various jerky categories.

### 3.4 Parameter tuning strategies and performance evaluation of machine learning algorithms in jerky type differentiation tasks

In the process of developing machine learning models, the selection of hyperparameters has a direct and significant impact on model performance. Hyperparameters are parameters set before the training of the model begins, and the predictive quality of the model largely depends on the selection and configuration of these hyperparameters (Hossain & Timmer, 2021). To investigate the effects of different hyperparameter tuning strategies on the accuracy of established classification models across two spectral bands, this paper analyzes five tuning-requiring algorithms, excluding NB.

The kernel function is the key hyperparameter in the SVM algorithm during training. Different kernel functions offer distinct advantages; appropriately selecting a kernel can simplify the complexity of dot product computations when SVM is applied to classify nonlinear data, thereby enhancing its ability to distinguish and amplify relevant features (Liu, Shen, & Wang, 2014). This study specifically considers the RBF kernel (default parameters), linear kernel, polynomial kernel, and Sigmoid kernel, all of which are commonly utilized in high-dimensional data modeling for non-destructive food testing (Lin, Ma, Wang, & Sun, 2023). In the ANN algorithm, the main hyperparameters pertain to the activation functions of the two hidden layers. By optimizing the choice of these activation functions, the model can incorporate necessary nonlinearity, allowing it to better learn and represent complex relationships (Rasamoelina, Adjailia, Sincák, & Ieee., 2020). The available activation functions include the ‘RelU’ (default parameter), ‘Exponential Linear Unit’ (ELU), Sigmoid function, and ‘hyperbolic tangent function’ (tanh). LR algorithm's main hyperparameters are the solver and dual parameters, both of which can be tuned (Saha et al., 2021). The solver parameter specifies the optimization algorithm, such as ‘L-BFGS’ (default) and ‘Liblinear’. The dual parameter affects computational efficiency and memory, applicable only with solver = ‘Liblinear’, with options True and False (default).For the DT and RF algorithms, the main hyperparameter adjusted is the criterion for measurinweg split quality, which includes ‘Gini’ index (default) and entropy.

As shown in Table 5, the detailed classification performance of five machine learning algorithms under different hyperparameter tuning strategies was presented. When the linear kernel function was utilized, the SVM model achieved high training and testing accuracies across both spectra, with test precision, recall, and F1 score exceeding 90 %, demonstrating high identification accuracy and robustness. For the ANN algorithm, the choice of activation function exhibited varying differentiation effects across different bands: compared to the default function, the ‘ELU’ activation function performed better in terms of recognition accuracy and model stability in the Vis-NIR band, whereas ‘Tanh’ activation function performed better in the SWIR band. For the LR model, all three tuning strategies achieved relatively ideal classification performance and model performance across both spectral bands, with the ‘Liblinear’ solver yieldeing the best testing results and exhibiting robust performance, while the dual setting had a minor impact on the testing performance in the Vis-NIR band. When the DT and RF algorithms were employed, both optimization strategies achieved 100 % training accuracy across the two bands. Although the RF algorithm improved prediction performance in the Vis-NIR band and the DT algorithm in the SWIR band, the average accuracy remained below 80 %, and the generalization capabilities of the models were poor. Notably, among all tuning strategies, the LR model and the SVM model with a linear kernel exhibited higher prediction accuracy than other algorithms. This suggests that these linear models are more effective in handling the spectral datasets.

**Table 5:** Identification results of each machine learning under different tuning optimisation scenario.

Machine learning algorithms	Parameter settings	10 × 10-Fold cross-validation re-averaged accuracy									
		400-1000 nm					900-1700 nm				
		Training accuracy	Test accuracy	Test precision	Recall	F1 score	Training accuracy	Test accuracy	Test precision	Recall	F1 score
SVM	Kernel = Rbf	87.99 %	79.24 %	86.18 %	80.48 %	80.43 %	91.83 %	81.12 %	97.17 %	83.86 %	84.26 %
	Kernel = Linear	98.21 %	89.29 %	93.40 %	91.26 %	91.06 %	99.48 %	95.29 %	94.47 %	93.24 %	93.02 %
	Kernel = Poly	67.86 %	55.86 %	65.94 %	55.52 %	54.77 %	75.02 %	65.76 %	77.01 %	63.00 %	64.77 %
	Kernel = Sigmoid	79.20 %	77.19 %	77.56 %	75.86 %	72.79 %	73.23 %	73.86 %	75.74 %	69.76 %	70.18 %
ANN	Activation = [Relu, Relu, Softmax]	87.38 %	84.54 %	79.05 %	79.13 %	75.99 %	91.21 %	88.85 %	80.90 %	84.33 %	80.30 %
	Activation = [Elu, Elu, Softmax]	90.82 %	88.67 %	84.34 %	85.63 %	83.00 %	93.81 %	91.86 %	86.56 %	85.70 %	85.42 %
	Activation = [Sigmoid, Sigmoid, Softmax]	81.65 %	79.86 %	70.67 %	77.33 %	70.90 %	83.86 %	82.52 %	75.43 %	78.07 %	73.10 %
	Activation = [Tanh, Tanh, Softmax]	91.42 %	86.62 %	80.84 %	80.77 %	78.67 %	93.88 %	92.62 %	86.42 %	86.20 %	85.34 %
LR	Solver = Lbfgs, Dual = False	98.11 %	91.92 %	89.67 %	92.55 %	89.52 %	99.31 %	94.07 %	91.96 %	89.43 %	89.58 %
	Solver = Liblinear, Dual = False	97.16 %	93.29 %	92.04 %	93.62 %	92.05 %	99.18 %	94.62 %	92.10 %	91.83 %	91.05 %
	Solver = Liblinear, Dual = True	96.72 %	93.29 %	92.04 %	93.61 %	92.05 %	98.88 %	95.26 %	92.10 %	91.83 %	91.05 %
DT	Criterion = Gini	100.00 %	69.95 %	70.00 %	72.20 %	66.96 %	100.00 %	60.73 %	66.74 %	68.80 %	64.71 %
	Criterion = Entropy	100.00 %	66.90 %	68.50 %	70.76 %	66.43 %	100.00 %	69.86 %	67.48 %	69.01 %	65.46 %
RF	Criterion = Gini	100.00 %	73.48 %	80.76 %	77.00 %	76.27 %	100.00 %	79.65 %	81.56 %	74.43 %	75.45 %
	Criterion = Entropy	100.00 %	77.00 %	80.13 %	77.05 %	76.50 %	100.00 %	76.49 %	82.24 %	76.48 %	77.54 %

## 4 Conclusion

This study was based on Vis-NIR and SWIR hyperspectral data, combined with mutiple machine learning algorithms to identify five types of commercial jerky. It explored the impact of different spectral bands, algorithm selection, and the application of multiple preprocessing and hyperparameter optimization methods on the identification of jerky types. The results indicated that normalization with randomization preprocessing improve the prediction accuracy of all models across both bands. Performance varied among algorithms across different bands. Among them, the LR model showed high identification accuracy across multiple jerky types, with prediction accuracy ranging from 85.78 % to 100.00 %, whereas other algorithms perform well only in specific types or bands. Furthermore, the spectral data of different bands exhibited significant impacts on jerky identificaion, and there are complementary information between bands. Specifically, the SWIR band outperforms the Vis-NIR band in identifying duck jerky, while in the Vis-NIR band, the prediction accuracy for chicken jerky is higher than that in the SWIR band (excluding ANN and DT). Hyperparameter optimization further enhances model identification performance, particularly in the case of non-linear SVM models, whose prediction accuracy was improved by employing linear kernel function settings, achieving prediction accuracy, recall, and F1 Score values above 90.00 %. On the whole, after optimization through normalization combined with randomization preprocessing and hyperparameter tuning strategies, the linear models (LR and SVM with a linear kernel setting) based on SWIR data can better identify between different types of jerky. The results of this study suggest that the combined application of hyperspectral imaging and machine learning presents a promising approach for the rapid, non-destructive differentiation of various jerky types. Given the complementary nature of spectral data across different bands and the existence of spectral bands closely related to sample color in Vis-NIR data, future research will explore the effective fusion of spectral data and images across both bands to enhance the precise identification of jerky varieties.

## CRediT authorship contribution statement

**Yuanxi Han:** Writing – review & editing, Writing – original draft, Investigation. **Liang Li:** Supervision, Funding acquisition, Conceptualization. **Siyuan Jiang:** Visualization, Resources. **Pengpeng Sun:** Validation, Software. **Wenliang Wu:** Validation, Supervision. **Zhendong Liu:** Supervision, Methodology.

## Declaration of competing interest

The authors declare that they have no known competing financial interests or personal relationships that could have appeared to influence the work reported in this paper.

## Data Availability

Data will be made available on request.
